# The mRNA puzzle: Intron retention under stress

**DOI:** 10.1093/plcell/koae093

**Published:** 2024-03-22

**Authors:** Laura Arribas-Hernández

**Affiliations:** Assistant Features Editor, The Plant Cell, American Society of Plant Biologists; Biology Department, University of Copenhagen, 2200 Copenhagen N, Denmark

Have you ever read one of those gamebooks from the 1980s in which the narrative splits into divergent paths, and you can choose your own adventure by skipping certain pages? Well then, you should consider that nucleated cells “invented” that concept some million years before that literary genre was ever popularized. In the nucleus, cells can literally choose their own mRNAs by deciding which parts of a transcript must be skipped (spliced out) and which parts shall be kept (Fig.). Like pieces in a puzzle, chunks of RNA can be combined in different ways, producing multiple messages from the same DNA. At the molecular level, this mechanism of alternative splicing (AS) is governed by the spliceosome, a multi-megadalton complex formed by 5 small nucleolar ribonucleoprotein particles (snRNPs), and over 200 accessory proteins (Wahl et al. 2009).

**Figure.**
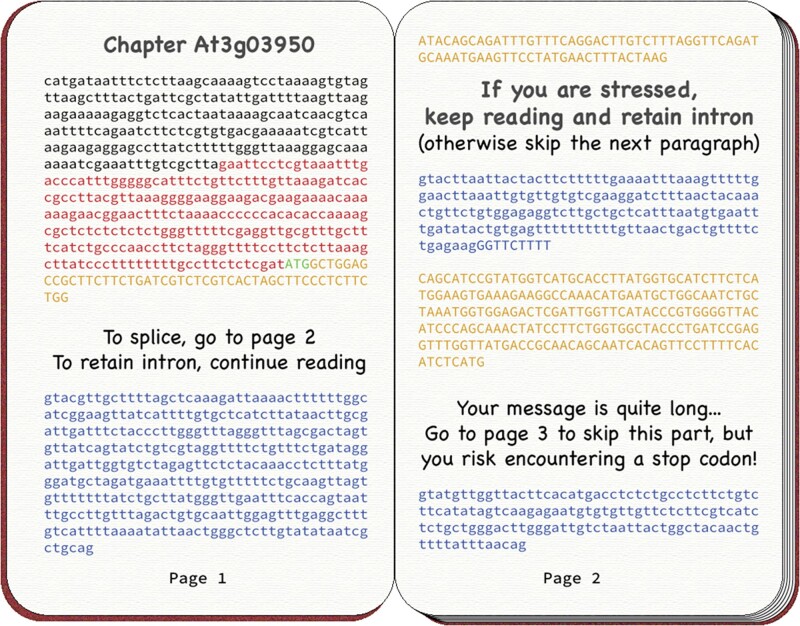
Metaphoric illustration of alternative splicing as a gamebook, by Laura Arribas-Hernández.

AS can be regulated, among other mechanisms, by the methylation of spliceosomal proteins catalyzed by protein arginine methyltransferases (PRMTs). These enzymes are well-studied in mammalian cells, as they can influence the progression of some types of cancer (Jarrold and Davies 2019). Although plants also make extensive use of AS to regulate essential physiological processes such as the circadian clock and various stress responses, the function of splicing factor methylation in plants is less well understood.

The best-characterized plant PRMT is Arabidopsis PRMT5, which catalyzes the symmetric dimethylation of arginine residues in several splicing factors, including the U6-snRNP constituent LSM4 (Sanchez et al. 2011). Plants lacking PRMT5 or LSM4 exhibit splicing abnormalities, and both types of mutants have comparable physiological defects (Sanchez et al. 2011). However, whether and to which extent PRMT5-mediated splicing factor methylation contributes to splicing regulation has not yet been determined. To fill this gap, Yamila Carla Agrofoglio, María José Iglesias et al. (Agrofoglio et al. 2024), in this issue, studied the direct effects of PRMT5-mediated arginine methylation of LSM4.

Agrofoglio et al. performed RIP-Seq to determine the identity of LSM4 targets in vivo, and analyzed transcriptomic data of *lsm4* knockout plants to identify transcripts whose abundance and/or splicing patterns are affected by loss of LSM4. Comparison of these datasets with transcriptomic data from *prmt5* mutants, published by the same authors (Mateos et al. 2023), revealed important overlaps between transcripts with defective splicing in the two mutants and those bound by LSM4. Although these results suggest that the influence of PRMT5 on AS is largely mediated by LSM4, they do not prove that the phenotypic defects of *prmt5* mutant plants are directly caused by lack of LSM methylation. To investigate that, the authors generated plants expressing an “unmethylable” version of LSM4 (LSM4^RxK^) in the *lsm4* knockout background. Unexpectedly, these transgenic lines demonstrated that LSM4 methylation is actually dispensable for growth in laboratory conditions, because expression of LSM4^RxK^ fully rescues *lsm4* defects in development and circadian rhythm (Agrofoglio et al. 2024).

Intrigued by the lack of developmental defects in *lsm4/LSM4^RxK^* plants, the authors set out to investigate the biological functions of PRMT5-mediated arginine methylation of LSM4. They performed RNA-seq experiments in *lsm4/LSM4^RxK^*, *lsm4/LSM4^WT^*, *lsm4*, and wild-type plants, analyzing the results through pairwise comparisons. These analyses revealed that (1) the overall effect on splicing caused by the absence of LSM4 is larger than that of its impaired methylation; (2) lack of LSM4 is detrimental to splicing, as it mostly lead to intron retention; (3) for ∼70% of the intron retention events observed in *lsm4* mutants, the unmethylable LSM4^RxK^ variant is better than LSM4^WT^ at restoring splicing, suggesting that methylation may inhibit the splicing-stimulating activity of LSM4 in some cases; and (4) there is a clear enrichment of genes related to abiotic stress and defense responses among those differentially spliced between LSM4^RxK^ and LSM4^WT^ (Agrofoglio et al. 2024). Hence, LSM4 methylation may be relevant only when plants are exposed to adverse conditions.

To investigate the possible role of LSM4 methylation in stress responses, Agrofolio et al. performed a series of functional assays, finding that the stress-signaling hormone abscisic acid (ABA) induces LSM4 methylation, whereas bacterial infection has an inhibitory effect. In line with these findings, *lsm4/*LSM4^RxK^ lines are hypersensitive to ABA or drought stress and exhibit enhanced resistance to bacterial infection, similar to *prmt5* mutants. Searching for LSM4 targets whose incorrect splicing could be responsible for the aberrant stress responses observed in the mutants, the authors identified several ABA-, drought-, and defense-response genes with different degrees of intron retention in *lsm4/*LSM4^RxK^ compared to *lsm4/*LSM4^WT^ lines. Follow-up studies will surely determine what is the relative contribution of these genes for the mutant phenotype.

## Dive Curated Terms

The following phenotypic, genotypic, and functional terms are of significance to the work described in this paper:

LSM4 Gramene: AT5G27720
LSM4 Araport: AT5G27720
